# Oxygen atoms are critical in rendering THP-1 leukaemia cells susceptible to cold physical plasma-induced apoptosis

**DOI:** 10.1038/s41598-017-03131-y

**Published:** 2017-06-05

**Authors:** Sander Bekeschus, Kristian Wende, Mohamed Mokhtar Hefny, Katrin Rödder, Helena Jablonowski, Anke Schmidt, Thomas von Woedtke, Klaus-Dieter Weltmann, Jan Benedikt

**Affiliations:** 1 Leibniz-Institute for Plasma Science and Technology (INP Greifswald) ZIK plasmatis, Greifswald, Germany; 2 Coupled Plasma-Solid State Systems, Faculty of Physics and Astronomy, Ruhr University Bochum, Bochum, Germany; 3 Basic Science Department, Faculty of Engineering and Technology, Future University in Egypt, Cairo, Egypt; 4 Institute for Hygiene and Environmental Medicine, University Medicine Greifswald, Greifswald, Germany

## Abstract

Cold physical plasma has been suggested as a powerful new tool in oncology. However, some cancer cells such as THP-1 leukaemia cells have been shown to be resistant towards plasma-induced cell death, thereby serving as a good model for optimizing plasmas in order to foster pro-apoptotic anticancer effects. A helium/oxygen radio frequency driven atmospheric plasma profoundly induced apoptosis in THP-1 cells whereas helium, humidified helium, and humidified helium/oxygen plasmas were inefficient. Hydrogen peroxide – previously shown as central plasma-derived agent – did not participate in the killing reaction but our results suggest hypochlorous acid to be responsible for the effect observed. Proteomic analysis of THP-1 cells exposed to He/O_2_ plasma emphasized a prominent growth retardation, cell stress, apoptosis, and a pro-immunogenic profile. Altogether, a plasma setting that inactivates previously unresponsive leukaemia cells is presented. Crucial reactive species in the plasma and liquid environment were identified and discussed, deciphering the complexity of plasma from the gas phase into the liquid down to the cellular response mechanism. These results may help tailoring plasmas for clinical applications such as oxidation-insensitive types of cancer.

## Introduction

Firmly connecting with redox biology, therapeutical effects of cold plasma-generated reactive molecules are investigated in the field of plasma medicine^1^. The strong advantage of plasma is the parallel deposition of different biologically active reactive species in a localized manner^2^. In the plasma gas phase, this includes for example hydroxyl radical, nitric oxide, and atomic oxygen^3^. In plasma-treated liquids, chemistry is further complexed, and typical molecules detected include superoxide anion, hydrogen peroxide, and peroxynitrite^4^. With adequate concentrations, these species can overwhelm the cells’ antioxidative response, effectively mediating pro-apoptotic redox signalling responses^5^. Many tumours display an inequity in their redox balance, rendering them more receptive towards oxidation-induced cell death compared to non-malignant cells^6^. Accordingly, a number of drugs have been tested in clinical trials aiming at further disturbing the redox-balance of cancers^7^, ultimately inducing apoptosis^8^. Thus, plasmas applications have been proposed to be a possible asset in oncology as well, as killing has been achieved for various types of cancers *in vitro* ^9–11^ and *in vivo* ^12–14^. Although primary monocytes are susceptible to plasma-induced apoptosis^15^, some malignant cells such as THP-1 leukaemia cells effectively withstand plasma-induced apoptosis^16^, and rather respond via growth deceleration^17^. They, therefore, serve as an ideal target to identify which parameters may render a plasma source more effective for oncological applications in oxidation-resistant cancer cells.

Different parameters can be adjusted to alter the plasma generation and thus the reactive species composition. For example, humidity in the feed argon gas was identified to play a highly critical role^18^. The humidification of a noble gas dramatically increase hydrogen peroxide (H_2_O_2_) production in the gas and liquid phase^19^ whereas addition of oxygen (O_2_) or nitrogen (N_2_) enhances the generation of reactive oxygen and nitrogen species (ROS, RNS)^4^, respectively. A helium (He)-driven atmospheric pressure plasma jet (µAPPJ) in its COST-jet version was used which has been characterized towards its physical and antimicrobial properties^20–22^. To investigate the biological effects of feed gas alterations, it was humidified and/or spiked with molecular oxygen (O_2_) with the idea of altering liquid chemistry, redox reactions, and subsequently eradication of the tumour target cells. In this study, we analysed cell morphology and counts, metabolic activity, apoptosis, and the THP-1 proteome on the one hand, and investigate the plasma-treated liquids on the other hand to determine the mechanism of action as function of the feed gas compositions.

## Results

### The feed gas composition-dependent THP-1 cell inactivation was mediated via apoptosis

**Figure 1:**
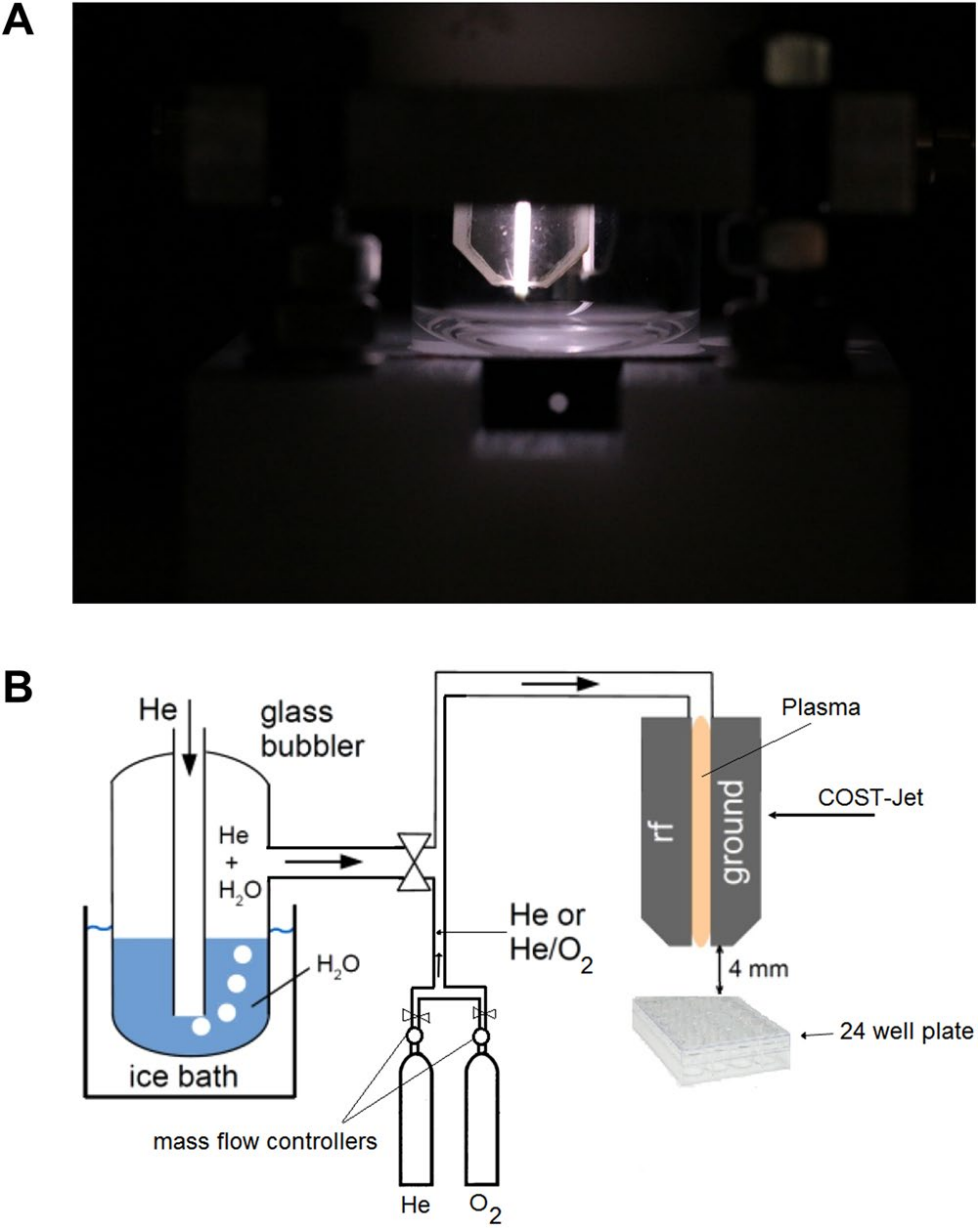
Image of the plasma source and its schematic overview. (**a**) Schematic representation of the plasma source with the bubbler and gas connections. Additionally, one well of a 24 well plate with the relative position of the jet are shown as well. (**b**) Photograph of the atmospheric pressure helium plasma jet (µAPPJ).

**Figure 2:**
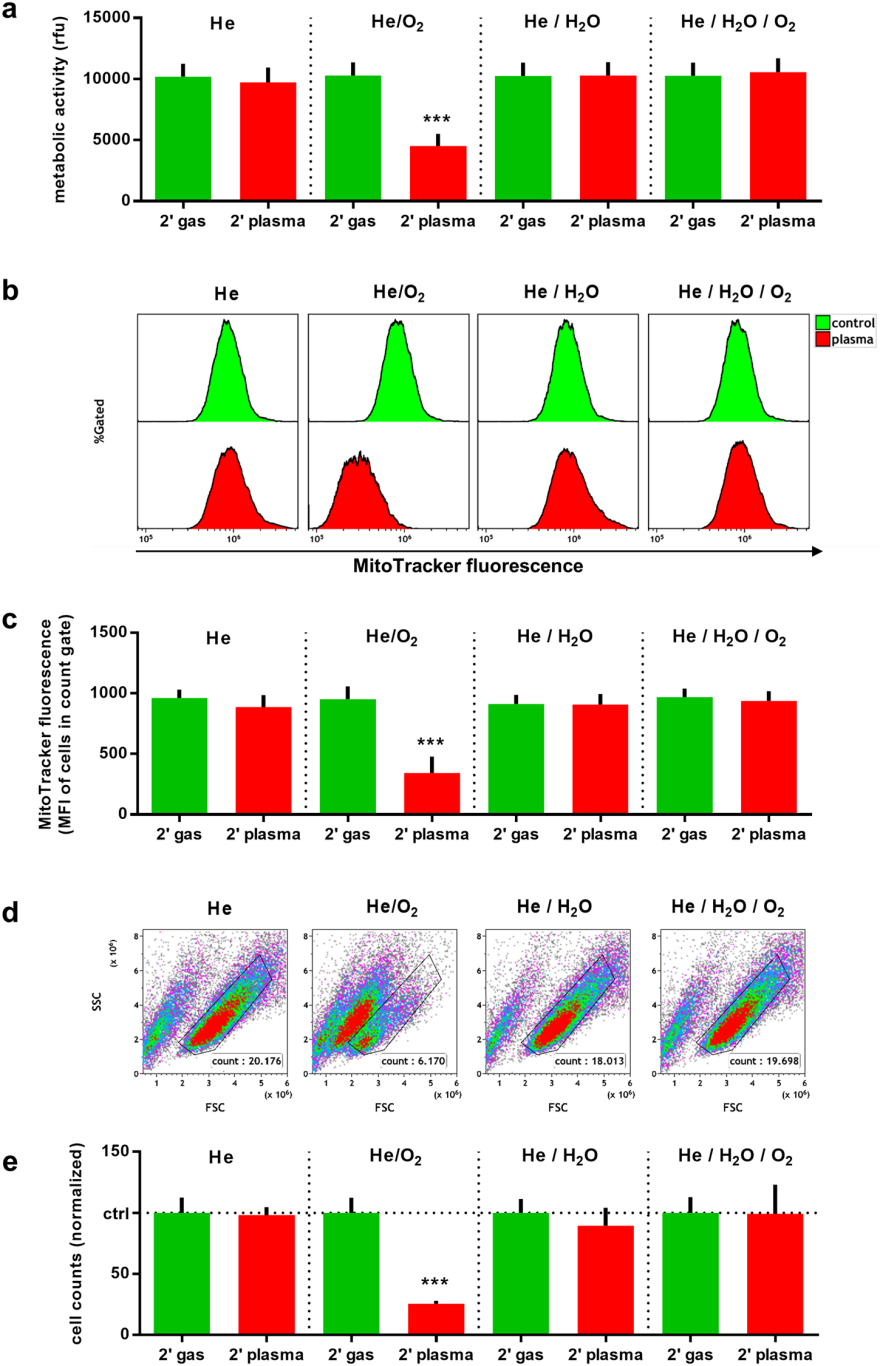
He/O_2_ but not He, He/H_2_O, or He/O_2_/H_2_O plasma inactivated THP-1 cells. THP-1 cells were exposed to cold physical plasma under different feed gas conditions, and assayed after 24 h. (**a**) Overall metabolic activity was significantly decreased in the He/O_2_ condition, as measured by resazurin to resorufin transformation. (**b**) Representative histograms of mitotracker fluorescence for each gas condition. (**c**) Mitotracker fluorescence quantification revealed significant reduction of mitochondrial activity in the He/O_2_ condition. (**d**) Representative FSC/SSC dot plots with the appropriate live and cell count gate. (**e**) Live cell count quantification demonstrated a significant decrease with the He/O_2_ condition. Data are one representative (**b**,**d**) or presented as mean + S.D. (**a**,**c**,**e**) of three experiments. Statistical differences (***p < 0.001) were determined using t-test.

**Figure 3:**
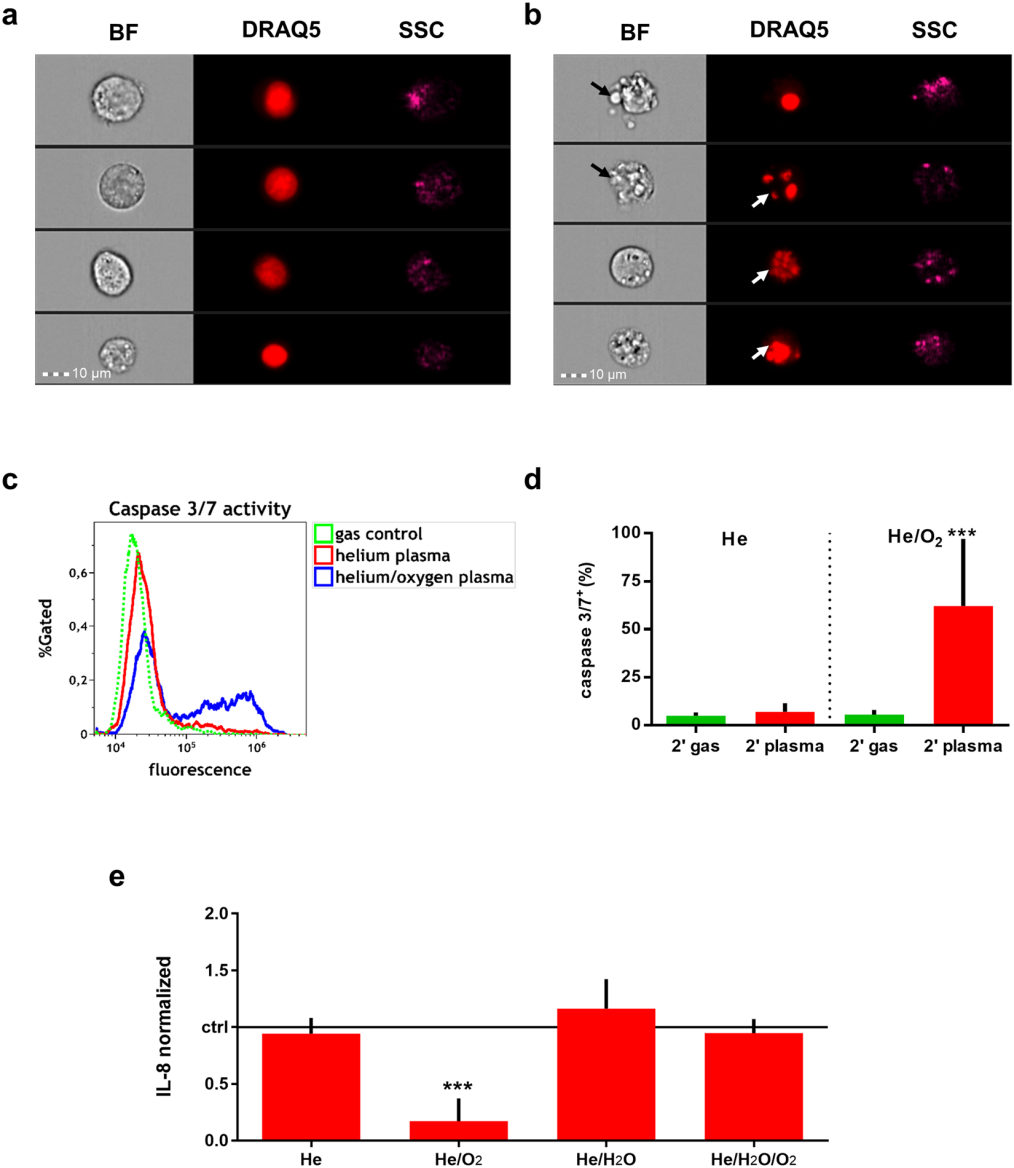
The mode of cytotoxicity of He/O_2_ plasma in THP-1 cells was apoptosis. (**a**) Control THP-1 cells showed a round morphology (BF, bright field, left array) with intact nuclei (DRAQ5, middle panel) and characteristic side scatter patterns (SSC, right panel) as measured using image cytometry. (**b**) Plasma-treated THP-1 cells displayed altered morphological features with membrane blebbing and vacuolization (BF) and segmented nuclei (DRAQ5). (**c**) Representative histograms of activated caspases 3/7 in plasma-treated cells. (**d**) Quantification of percent of cells being positive for active caspases 3/7 with a significant increase in He/O_2_ but not the He plasma condition. (**e**) THP-1 cell culture supernatant was investigated for IL-8 24 h after exposure to plasma generated using different feed gas compositions. Final concentrations where normalized to each gas-treated control, respectively. He/O_2_ but not any other gas plasma significantly decreased IL-8 concentrations in the supernatant of THP-1 cells. Data are one representative (**a**,**b**,**c**) or mean (**d**,**e**) + S.D. of three experiments. Statistical analysis was determined using the t-test (***p < 0.001).

THP-1 cells are known to strongly persist oxidative stress-induced killing. The aim of this work was to identify a feed gas composition that may foster cold plasma-induced eradication of these cells to substantiate its potential use in tumour therapy. THP-1 cells were exposed to a radiofrequency driven plasma jet utilizing He, He/0.6% O_2_, He/0.15% H_2_O, or He/0.6% O_2_/0.15% H_2_O as feed gases (Fig. 1), and were assayed 24 h later. The conversion of the resazurin to its fluorescent product resorufin via nicotinamide adenine dinucleotide phosphate reduction equivalents identifies the overall metabolic activity of given number of cells. A significantly decreased metabolism could be observed in cells treated with He/O_2_ plasma but not with any of the other feed gas conditions used (Fig. 2a). Flow cytometry was performed to determine the mitochondrial activity on a single cell level using mitotracker orange dye (Fig. 2b). Highly fluorescent once incorporated into mitochondria, its fluorescence decreases if the mitochondrial membrane potential is lost^23^, which indicates cell death. Only cells exposed to the He/O_2_ plasma showed a significant impairment (Fig. 2c) which may have contributed to the overall decreased metabolic activity seen with the resazurin dye. It was asked next whether this finding may also be attributed to a decrease in total cell numbers in the appropriate forward scatter (FSC)/side scatter (SSC) gate (Fig. 2d). Again, only the He/O_2_ plasma condition led to a significant reduction of cells (Fig. 2e). The data of viable cell counts (Fig. 2e) and metabolic activity (Fig. 2a) are not congruent. This may be a consequence of apoptosis being an active and energy consuming process^24^ together with the observation that plasma treatment may increase activity in viable THP-1 cells^16^. Altogether, He/O_2_ but no other plasma conditions efficiently reduced THP-1 cell metabolic activity by inducing a down-modulation of mitochondrial activity and via reduction of total cell counts. In none but the He/O_2_ condition, cytotoxicity could be observed (Fig. 2). We, therefore, assessed morphological features of control (Fig. 3a) and He/O_2_ plasma-treated (Fig. 3b) THP-1 cells after 24 h using image cytometry. Treated but not control cells showed features of apoptosis such as membrane blebbing, nuclear defragmentation with similar side-scatter profiles of dead cells as seen with traditional cytometry (Fig. 2d). To further validate the apoptotic process, THP-1 cells were stained for activated executioner caspases 3/7 (Fig. 3c), and He/O_2_ but not He plasma-treated cells were shown to be significantly positive (Fig. 3d). Linking to these apoptotic events, concentrations of interleukin (IL)-8 were significantly decreased in He/O_2_ plasma-treated samples as well (Fig. 3e).

### THP-1 cell killing in the He/O_2_ condition was mediated via a non-H_2_O_2_ mechanism

**Figure 4:**
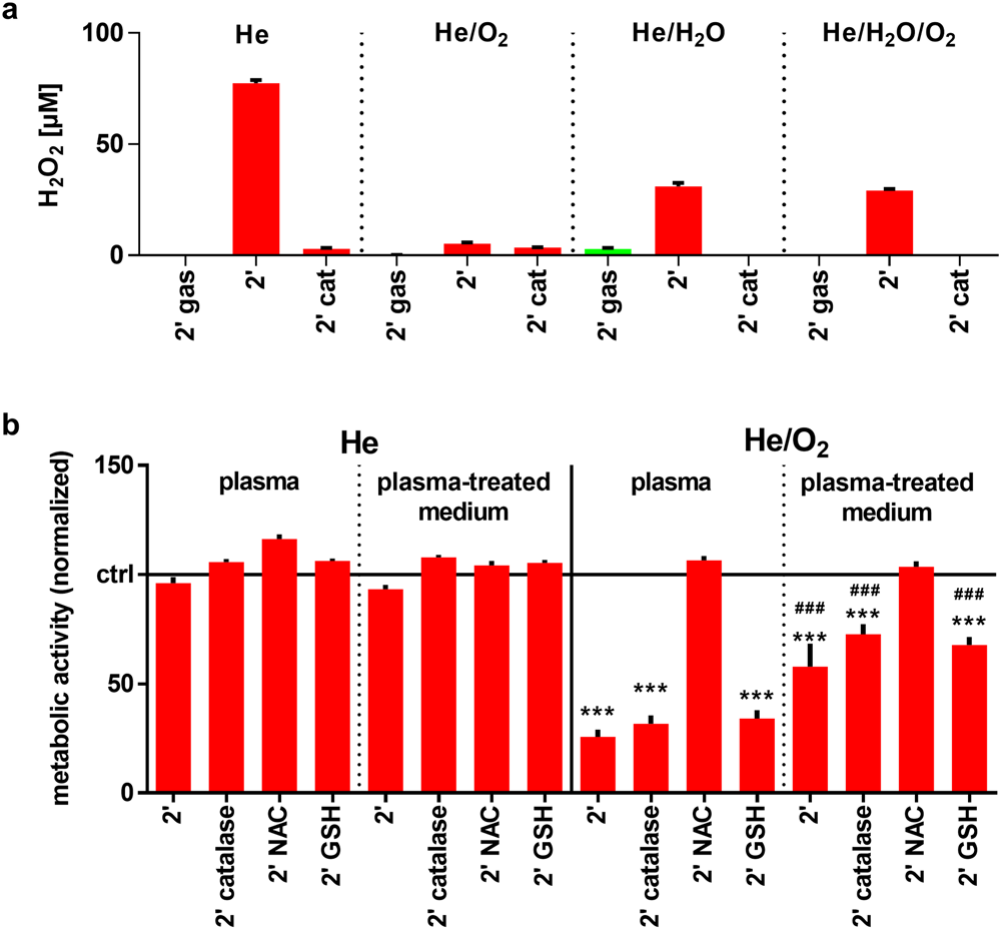
Not H_2_O_2_ but another long-lived oxidant was responsible for THP-1 cell killing. (**a**) H_2_O_2_ was assessed in plasma-treated cell culture medium using different feed gas conditions, and He plasma showed the highest production with catalase scavenging H_2_O_2_ in any feed gas condition. (**b**) THP-1 cells were either exposed to He or He/O_2_ plasma or to He or He/O_2_ plasma-treated medium which was immediately added afterwards using different feed gas conditions and in presence or without of either catalase, NAC, or GSH. Cells were assayed for metabolic activity 24 h later, and showed a significant reduction (***) in presence of catalase or GSH but not NAC. This effect was less pronounced and differed significantly (###) for plasma-treated medium compared to direct treatment in the He/O_2_ condition. Data are presented as mean + S.D. of two experiments. Statistical differences (***/^###^ *p* < 0.001) were determined using 1-way ANOVA within each gas condition and plasma treatment regimen, and t-test to compare individual values for a single treatment regimen within each gas condition.

**Figure 5:**
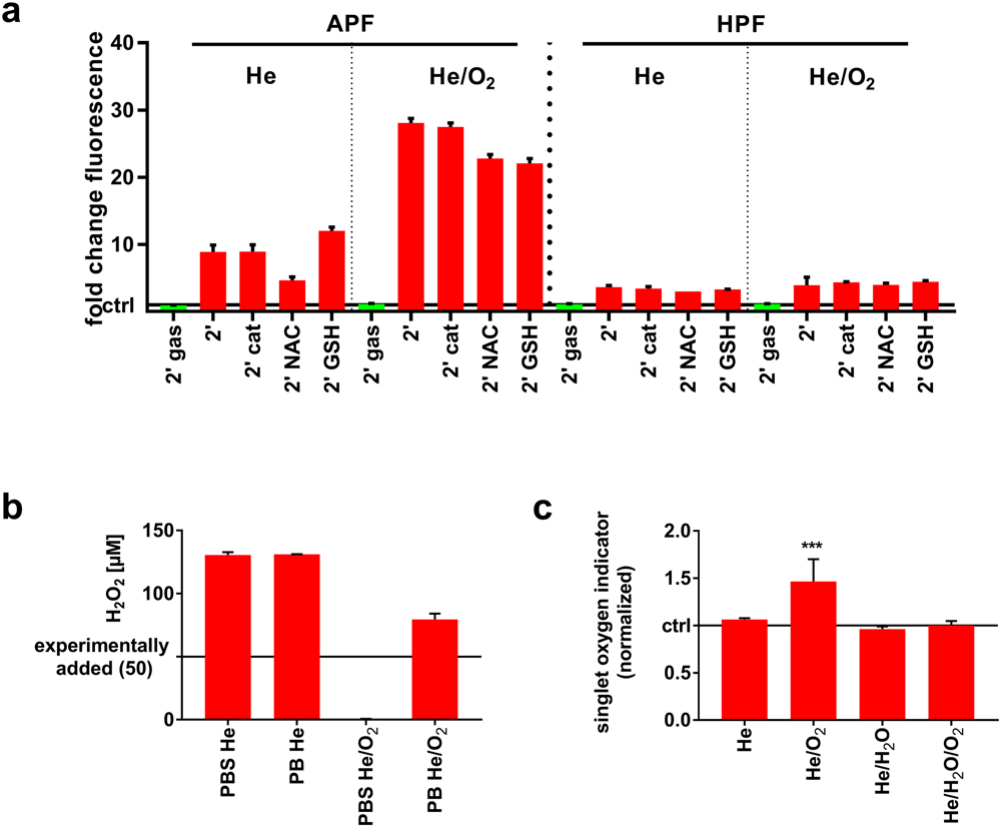
He/O_2_ plasma generated hypochlorous acid in the liquid via gaseous phase oxygen species. (**a**) Cell culture medium as loaded with either APF or HPF. Scavengers were added or not, solutions were plasma-treated using either He or He/O_2_ as feed gas, and fluorescence of the redox dyes was acquired which was normalized to each respective gas control. APF in general gave a stronger fluorescence signal which was increased in the He/O_2_ condition but not using the He plasma. NAC partially scavenged signals. (**b**) Fifty micromolar H_2_O_2_ was added to PBS and chloride-free phosphate buffer (PB). Solutions were plasma-treated using either He or He/O_2_ plasma. The latter scavenged experimentally added H_2_O_2_ in PBS but not PB. (**c**) Singlet oxygen sensor green was added to cell culture medium which was subsequently exposed to either He, He/O_2_, He/H_2_O, or He/H_2_O/O_2_ plasma. Fluorescence was acquired and normalized to untreated gas control, and only He/O_2_ plasma differed significantly. Data are presented as mean + S.D. of two experiments. Statistical difference (***p < 0.001) was determined using t-test.

THP-1 cells showed a high susceptibility towards He/O_2_ but not He, He/H_2_O, and He/H_2_O/O_2_ plasma. H_2_O_2_ was previously shown to be an important mediator in plasma cytotoxicity^25^. Yet, its concentration was decreased in the He/O_2_ plasma toxic condition compared to the helium plasma (Fig. 4a). Additionally, the enzymatic antioxidant catalase, a potent scavenger of H_2_O_2_^26^, did not inhibit the cytotoxic effect of He/O_2_ plasma (Fig. 4b). H_2_O_2_ contributed, therefore, unlikely to the killing mechanism. To test whether the responsible reactive molecule would be rather short or long lived, THP-1 cells were exposed to plasma-treated medium in presence of absence of the potent thiol-containing non-enzymatic antioxidants N-acetyl-cysteine (NAC) or glutathione (GSH). GSH is among the most abundant proteins in cells, and its oxidized dimer GSSG in readily replenished in cells by glutathione reductase^27^. Intracellularly, NAC serves as a GSH precursor, and its extracellular antioxidant activity is superior to that of GSH^28^. Similar to direct treatment, helium plasma-treated liquid did not affect cell viability (Fig. 4b). On the contrary, He/O_2_ plasma-treated liquid exhibited significant cytotoxic activity, although to a significantly lesser extent compared to direct plasma exposure (Fig. 4b). The toxic effects were abrogated in the presence of NAC but not glutathione GSH. To identify the species possibly entangled in this reaction, two dyes (3′-(*p*-aminophenyl) fluorescein, APF; or 3′-(*p*-hydroxyphenyl) fluorescein, HPF) were plasma-treated. In contrast to HPF, APF has been reported to distinguish for hypochlorous acid (HOCl)^29^ and has been used and validate throughout several studies in cells and liquids^30–32^. With APF but not HPF, fluorescence was markedly increased in the He/O_2_ compared to the He plasma condition (Fig. 5a). NAC and GSH but not catalase partially protected APF from oxidation. These experiments suggested HOCl to be generated in the liquid by He/O_2_ plasma treatment. HOCl is known to scavenge H_2_O_2_. A known concentration of H_2_O_2_ was experimentally added to PBS and chloride-free phosphate buffer and both solutions were exposed to plasma (Fig. 5b). With He plasma, H_2_O_2_ concentrations increased above that added concentration in both solutions as expected. This was also obtained for He/O_2_ plasma treating chloride free-buffer, as HOCl cannot be generated without chloride ions. However, He/O_2_ treatment of chloride-containing buffer fully consumed the experimentally added H_2_O_2_, further suggesting the production of HOCl under this condition. It was hypothesized that HOCl in chloride containing liquids can derive especially from atomic oxygen but also singlet oxygen coming from the plasma gas phase or generated in the liquid. Using a fluorescent singlet oxygen sensor, a moderate but significant increase in singlet oxygen in the liquid was found only in the pro-apoptotic He/O_2_ plasma but no other gas condition (Fig. 5c), underlining that changes of reactive components in that particular plasma composition were crucial for the observed effects.

### Pro-apoptotic and growth deceleration proteins regulated in He/O_2_ plasma-treated cells

THP-1 cells showed a high susceptibility towards He/O_2_ plasma. Using proteomic technology, up or down-regulation of proteins in the cellular fraction of THP-1 cells 4 h after plasma treatment with either He or He/O_2_ with or without the presence of NAC was assessed against gas-treated control cells. Gene ontology analysis identified binding (37%), catalytic activity (33%), structural molecule activity (15%), transporter activity (5%), and receptor activity (4%) as main molecular function categories of proteins that were significantly regulated in He/O_2_ plasma-treated compared to gas-treated control cells. Six representative significantly up or down-regulated proteins for the He/O_2_ treated cells are given (Table 1) together with the regulation in the other three samples types. Addition of NAC abrogated the regulation of any protein in plasma-treated samples to the level of untreated gas controls. This corroborates the findings with metabolic activity where NAC fully protected against toxic He/O_2_ plasma activity on THP-1 cells. Consistent with flow cytometric active caspase 3 detection, an upregulation (+11.1-fold) of this protein was also determined in the cellular fraction using mass spectrometry. Other proteins involved in apoptosis, transport or cell growth were also strongly regulated, for example, A-kinase anchor protein 8-like, Exportin-T, Telomeric repeat-binding factor 2-interacting protein, Cell and growth-regulating nucleolar protein (LYAR), and U4/U6.U5 tri-SNRNP -associated protein 1. A pro-immunogenic protein (Squamous cell carcinoma antigen recognized by T-cells 3) was increased as well.

**Table 1:** THP-1 killing by plasma was accompanied by alterations in protein expression involved in apoptosis and growth retardation.

Protein ID	Protein name	He	He/O_2_	He + NAC	He/O_2_ + NAC
P42574	Caspase 3	+1.9	+11.4	+1.1	+1.1
Q15020	Squamous cell carcinoma antigen recognized by T-cells 3	+1.5	+11.1	+1.3	−1.2
Q99459	Cell division cycle 5-like protein	−1.1	+4.1	+1.2	−1.3
P55769	U4/U6.U5 tri-SNRNP-associated Protein 1	−1.2	+2.6	−1.2	1.0
Q09472	Histone acetyltransferase p300	−2.5	+2.3	−1.1	−1.3
Q9NYL2	Mitogen-activated protein kinase kinase kinase MLT	+1.1	+2.1	+1.1	−1.1
P46013	Antigen Ki-67	−4.5	−2.3	1.0	−1.3
Q9NX58	Cell growth-regulating nucleolar protein	−1.8	−2.3	−1.1	−1.1
Q86WC6	Protein phosphatase 1 regulatory subunit 27	−1.1	−3.4	+1.3	+1.5
Q9NYB0	Telomeric repeat-binding factor 2-interacting protein 1	+1.2	−3.4	+1.1	−1.2
O43592	Exportin-T	−3.4	−3.4	−1.1	1.0
Q9ULX6	A-kinase anchor protein 8-like	−11.1	−8.3	+1.2	1.0

In presence of NAC (2 mM) or vehicle control, THP-1 cells were either left untreated or exposed to either He or He/O_2_ plasma. Four hours after treatment, cells were lysed, and proteins were analysed with a proteomic approach using LC/MS. Six representative positively and negatively regulated proteins with at least 2-fold regulation are given in the table for all four treatment regimes. Data sorting is shown in a descending manner for He/O_2_ plasma results.

## Discussion

Cold physical plasma-derived reactive species are a promising new tool in oncology^33^. However, some tumours are highly resistant to oxidative stress-induced cell death^34^. By modulating the reactive species output, it is, therefore, important to identify plasma-settings that also sensitize these cancer cells to apoptosis. Humidification of the carrier gas was previously described to potentiate HO^.^ and H_2_O_2_ generation of an atmospheric pressure argon plasma jet manifold^18^. These plasma-derived molecules are strong anticancer agents^35^. Yet, THP-1 cells resist high H_2_O_2_ concentrations and long plasma treatment times^16, 17, 36^. By admixing low amounts of oxygen to the feed gas of a helium plasma jet, a redox route was identified that is particularly effective in THP-1 cell killing. In line with classical hallmarks of apoptosis, treated cells showed membrane blebbing and executioner caspase 3 activation^37^. Interestingly, mitotracker orange fluorescence was also decreased in the viable cell portion. This argues for a destabilized mitochondrial membrane potential^23^ i.e. at least partial damage in He/O_2_ plasma-treated cells. The reduction in viability was also reflected by a decrease in constitutively expressed^38^ IL-8. This is interesting as oxidant exposure with only moderate apoptotic effects strongly increased IL-8 release in THP-1 cells^16, 39^ and other myeloid cells undergoing plasma-induced cell death^40^. IL-8 release is subject to redox control^41^ and a key molecule in regulating inflammation in wound healing^42^ and cancer^43^. Its release by e.g. monocytes/macrophages attract neutrophils to clear infection but also spur inflammation^44^.

Using proteomics, we identified proteins involved in growth retardation and cell death in He/O_2_ plasma-treated THP-1 cells. Protein phosphatase 1 regulatory subunit 27 negatively regulates phosphatases, and its significantly decreased recovery (−3.4-fold) reasons for sensation of cell stress resulting in target protein phosphorylation^45^. Cell and growth-regulating nucleolar protein was significantly increased (+4.1-fold). It is a crucial regulator of ATR-Chk1^46^ which is central in sensing redox stress as well^47^. This hampers cell cycling that is reflected by significant down-regulation of A-kinase anchor protein 8-like (−8.3-fold) which initiates initial phases of DNA replication^48^. Vice versa, U4/U6.U5 tri-SNRNP-associated protein 1, which is known to induced cell cycle arrest^49^, was upregulated (+2.6-fold). Paralleling growth deceleration, Exportin-1, important in exporting tRNAs from the nucleus to support protein translation and overexpressed in many tumors due to high proliferation rates^50^, was significantly down-regulated (−3.4-fold). Similarly, the down-regulation of Telomeric repeat-binding factor 2-interacting protein 1 (−3.4-fold) suggest growth retardation as it phosphorylates the inhibitor of NfκB thereby fostering cellular activation^51^. The protein LYAR is central for cellular growth^52^ and was significantly reduced (−2.4-fold) in plasma-treated cells. Vice versa, the proliferation marker Ki-67 was found to be decreased (−2.3-fold) whereas levels of cell death executioner caspase 3 drastically increased (+11.4-fold), in line with the flow cytometry experiments. This is underscored by elevated levels of Mitogen-activated protein kinase kinase kinase MLT (+2.1-fold), an upstream regulator Jnk and p38 known to be pro-apoptotic also in other cancer cells^53^. Interestingly, Histone acetyltransferase p300, a current target for anticancer therapy^54^, was increased in He/O_2_ plasma-treated THP-1 cells. Also of potential therapeutic value is the massive elevation of Squamous cell carcinoma antigen recognized by T-cells 3 (SART3) which has been used to potentiate T cell responses in vaccination trials in cancer patients (+11.1-fold)^55^. It is overexpressed in the majority of adenocarcinomas and squamous cell carcinomas in different tissues as well as in leukemia but not non-malignant cells^56^. Several reports conclude that SART3 is among a few targets found in many cancers with value in immunotherapy^57–59^. Altogether, the toxic plasma condition in THP-1 leukemia not only induced pro-apoptotic proteins but also suggested an enhanced immunogenicity of treated cells.

Significant cell responses were found to be present only in the He/O_2_ but not in any other feed gas condition. Although not necessarily the single responsible agent^60–62^, H_2_O_2_ has been determined to be central in plasma-mediated cytotoxicity in a number of studies^63–66^. Here, its direct effects were negligible as results with catalase suggest. Instead, we suspect HOCl to be the active cytotoxic agent for a number of reasons. NAC, an efficient scavenger of HOCl^67^, abrogated the He/O_2_ plasma’s cytotoxicity. HOCl is also a known scavenger of H_2_O_2_^68^. A full decrease of experimentally added H_2_O_2_ was only apparent in the toxic He/O_2_ but not the non-toxic He plasma conditions. Moreover, scavenging was abolished in the absence of chloride which is apparently essential for the generation of HOCl in liquids^69^. Both APF and HPF are reported to sense HO^.^ and ONOO^−^^70^. APF but not HPF is sensitive for HOCl^29^ and used to detect halogenated acids such as HOCl in phagocytes^32^ and leukemic cells^71^. He/O_2_ but not He plasma increased APF but not HPF fluorescence. These results suggest the presence and activity of HOCl, although a previous report using another plasma jet suggested RNS to be important in THP-1 cell inactivation^72^. Another possibility is that HOCl acted on cell culture proteins that then have mediated cytotoxic effects. In mammals, HOCl is physiologically generated by myeloperoxidase primarily expressed by neutrophils^73^. HOCl especially serves to destruct phagocytosed bacteria^74^. It is also present extracellularly, for example as a by-product of neutrophil extracellular traps generated during infection^75^. HOCl is highly reactive and capable of inactivating protease inhibitors and lysozyme^76^. Thus, it has an important role in fighting bacteria and spurring inflammation. Nonetheless, HOCl is readily detoxified by cellular thiols^77^, albumin^78^, and cross linking of biological molecules via non-disulfide bonds^79^, effectively limiting its damage to host cells.

The addition of oxygen to dry helium feed gas was found to be central for plasma to inactivate THP-1 cells in a HOCl-dependent manner. HOCl generation is thought to be mediated via atomic oxygen (1):

1 $${\rm{O}}+{{\rm{Cl}}}^{-}\to {{\rm{OCl}}}^{-}$$

Underlining this notion^80^, O densities in the gas phase of this plasma source have been reported to be markedly elevated in the effective He/O_2_ plasma (10^15^ cm^−3^)^81^ compared to He and He/H_2_O plasma (10^13^ cm^−3^)^82^. Hypochlorous acid can be formed via the reaction of O with Cl^−^, and our experiments using chloride-free buffer support the significance of this route in our study. In humidified feed gas, the generation of OH· and not O is dominant^83^ which is reflected in the relatively H_2_O_2_ resistant THP-1 cells^17^ not being strongly affected with these plasma conditions in the present study. Importantly, O shows a good solubility in liquids as previous work using phenol as target suggest^83^ which makes its important role in HOCl generation plausible.

A further source of reactivity in the He/O_2_ gas mixture could be other excited molecular oxygen species, especially the singlet delta oxygen O_2_(^1^Δ_g_) with a gas phase density of around 4 × 10^14^ cm^−3^ with similar power and gas conditions as used in this work^84^. Yet, its density was previously found to decrease with increasing O_2_ concentration (due to fast quenching in collisions with O atoms in the gas phase), limiting its possible impact. Nonetheless, in He/O_2_ plasma but no other feed gas condition, singlet delta oxygen was modestly increased in treated medium containing L-histidine, which was technically suboptimal as it can quench singlet oxygen^85^. According to the following net reaction (2):

2 $${\rm{HOCl}}+{{\rm{H}}}_{2}{{\rm{O}}}_{2}\to {{\rm{Cl}}}^{-}+{{\rm{H}}}^{+}+{{\rm{H}}}_{2}{\rm{O}}+{{\rm{O}}}_{2}({}^{1}{\rm{\Delta }}_{g}),$$

Hypochlorous acid oxidizes hydrogen peroxide yielding chloride ions^86^, water, and singlet delta oxygen (O_2_(^1^Δ_g_)). Together with the absence of H_2_O_2_ this notion further emphasizes the role of HOCl as an active agent under the discussed circumstances. Consecutively, O_2_(^1^Δ_g_) created in this secondary reaction may contribute to the observed effect, but considering its very short lifetime in aqueous, neutral liquids (few µs) compared to HOCl (several minutes)^87^, to a limited extent only. Notably, ozone production also increases 10-fold in the toxic oxygen setup^81^. Yet, and in contrast to treating dry surfaces^88^, modelling studies grant only a minor role to ozone in the liquid^89^ as it dissolves badly and no reactions with phenols could be detected^83^.

## Conclusion

Cold physical plasma has been suggested to be an option for cancer therapy, but some cancers are highly resistant against plasma-induced peroxide stress. By modulating the feed gas of a helium-driven plasma jet by the addition of oxygen, it was demonstrated that the chemistry can be tuned towards other species such as hypochlorous acid. With similar treatment times, this condition severely hampered cell activity and growth by inducing apoptosis whereas plasma treatments dominated by hydrogen peroxide did not. Additionally, immune stimulatory proteins were found to be elevated. Understanding the biological impact of gas and liquid phase plasma chemistry will help improving the plasmas’ efficacy in order to tailor them to the therapeutic applications’ needs.

## Materials and Methods

### Plasma Source

The COST jet, a proposed reference jet in the European Cost action MP1101 to compare effects of plasma treatments among different laboratories, was used as plasma source (Fig. 1a)^20^. Helium (99.9999%; Air liquide, France) at a flow of 1.4 slm was used as feed gas. In some experiments, it was admixed with either 0.6% oxygen or humidified helium with approximately 1450 ppm of H_2_O, or both. Humidification was achieved by bubbling 0.3 slm of helium flow through 400 ml of double distilled water and mixing it with 1.1 slm of dry Helium to obtain the total flow of 1.4 slm. (Fig. 1b). Calibrated, USB-controlled flow controllers (MKS instruments, Germany) were used to set and monitor all feed gas conditions.

### Cell culture and plasma treatment

The leukaemia cell line THP-1 (ATCC TIB-202, Germany) were cultured in fully supplemented RPMI1640 cell culture medium containing 10% fetal bovine serum, 2% glutamine, and 1% penicillin-streptomycin (all Sigma, Germany) in an incubator (Binder, Germany) at 37 °C, 95% relative humidity, and 5% CO_2_. For plasma-treatment, 1 × 10^5^ cells in 250 µl of medium were seeded in each well of a 24-well plate (Nunc, Denmark). To some wells, the H_2_O_2_ scavenging enzyme catalase (20 µg/ml), the antioxidant GSH (1 mM) or the antioxidant NAC (2 mM) was added (all Sigma). Cells were directly exposed to either each plasma condition or their respective gas controls (plasma off). Alternatively, 1 × 10^5^ cells in 125 µl of cell culture medium received 125 µl of 250 µl medium (indirect approach) plasma-treated for the double amount of time for comparison. Following exposure to plasma or plasma-treated liquid, cells were returned to the incubator for 20 h or 24 h depending on the subsequent analysis.

### Cellular analysis and viability

THP-1 cells were investigated on a number of properties, such as cell morphology, total metabolic activity, total cell counts, individual mitochondrial activity, and caspase 3/7 activity. For assessment of morphology 24 h after treatment, THP-1 cells were collected and stained with DRAQ5 (BioLegend, UK) prior to image acquisition using an *ImageStream Mark X* (Merck-Millipore, USA). Cells representative for viable and apoptotic events were analysed using *Ideas 6*.*2 software* (Merck-Millipore). For assessment of total metabolic activity 20 h after treatment, resazurin (Alfa Aesar, USA) was added to the wells, and cells were incubated for another 4 h. Supernatants were transferred to 96-well plates and fluorescence was acquired using a microplate reader with λ_ex_ 530 nm and λ_em_ 590 nm (Tecan, Switzerland). To assess mitochondrial activity 24 h after treatment, cells were stained with 500 nM mitotracker orange (CMXRos; life technologies, USA) for 20 min at 37 °C, and mitotracker fluorescence of cells in the respective gate was acquired using an *CytoFlex* cytometer (Beckman-Coulter, USA). Mitotracker orange is retained in mitochondria due to their chloromethyl group forming a covalent bond with thiols^90^. Moreover, the dye fluoresces to a lesser extend upon mitochondrial damage and membrane depolarization^23^. For total cell counts, cells were aliquoted from the 24-well plates into 96-well plates 24 h after treatment and counted with an *attune* flow cytometer (Applied Biosystems, USA) capable of measuring absolute particle concentrations. To assess apoptosis 24 h after treatment, THP 1 cells were collected, washed, and stained for 30 min at 37 °C with caspase 3/7 indicator (life technologies). The percentage of cells staining caspase positive was quantified using the *CytoFlex* cytometer.

### Redox-sensitive probes

Fully supplemented RPMI1640 medium was loaded with 1 µM singlet oxygen sensor, or PBS was loaded with 1 µM of either the fluorescent redox indicators APF or HPF (life technologies). Both APF and HPF can be oxidized by hydroxyl radicals and peroxynitrite but not hydrogen peroxide whereas only APF is sensitive towards hypochlorous acid^29^. Two-hundred and fifty microliter was added to each well of a 24-well plate, and wells either received plasma treatment or were left untreated. Subsequently, the liquid was aliquoted into 96-well plates, and fluorescence was acquired using a microplate reader with λ_ex_ 485 nm and λ_em_ 535 nm (Tecan). In order to quantify hydrogen peroxide (H_2_O_2_), plasma-treated cell culture medium or plasma-treated chloride-free phosphate buffer was incubated with amplex ultra red (life technologies) according to the vendor’s instructions, and subsequently quantified against an H_2_O_2_ standard using a Tecan microplate reader with λ_ex_ 530 nm and λ_em_ 590 nm. Alternatively, cell culture medium was spiked with a known concentration of H_2_O_2_ and the scavenging activity of plasma-introduced reactive components on H_2_O_2_ was assessed in a similar manner.

### Interleukin 8 and global protein expression

Twenty-four hours after plasma treatment, IL-8 in supernatants of THP-1 cells was quantified using ELISA (BioLegend), and concentrations were normalized to each respective gas control. Four hours after treatment, either eight replicates of untreated or plasma-treated THP-1 cells were pooled into micro-centrifuge tubes, washed, and stored at −80 °C. Global protein expression was carried out as previously described^91^. Briefly, peptides were separated by nano-liquid chromatography (Dionex Ultimate 3000; PepMap RSLC column, 75 µm ID/15 cm), and eluates were ionized by electrospray ionization and analysed by high-resolution mass spectrometry (QExactive, Thermo, USA) mass spectrometer. Data processing was done using Proteome Discoverer 1.4 software (Thermo). Protein candidates were selected upon their involvement in pathways of metabolisms and proliferation as well as on statistical criteria (≥ ± 2.0-fold expression). Data were also analysed with Ingenuity Pathway Analysis software (IPA, Qiagen) and free web based applications (PANTHER and Universal Protein Resource).

### Statistics

Statistical analysis was carried out using *prism 7*.*02* (graph pad software, USA). A significance level of α = 0.05 was set for all statistical analysis, and significantly different data were marked with asterisks (*p < 0.05; **p < 0.01; ***p < 0.001).
